# [(18)F]Fluoroethyltyrosine- positron emission tomography-guided radiotherapy for high-grade glioma

**DOI:** 10.1186/1748-717X-3-44

**Published:** 2008-12-24

**Authors:** Damien C Weber, Thomas Zilli, Franz Buchegger, Nathalie Casanova, Guy Haller, Michel Rouzaud, Philippe Nouet, Giovanna Dipasquale, Osman Ratib, Habib Zaidi, Hansjorg Vees, Raymond Miralbell

**Affiliations:** 1 Department of Radiation Oncology, Geneva University Hospital, CH-12011 Geneva 14, Switzerland; 2 Department of Nuclear Medicine, Geneva University Hospital, CH-12011 Geneva 14, Switzerland; 3 Unit of Clinical Epidemiology and Statistics, Geneva University Hospital, CH-12011 Geneva 14, Switzerland

## Abstract

**Background:**

To compare morphological gross tumor volumes (GTVs), defined as pre- and postoperative gadolinium enhancement on T_1_-weighted magnetic resonance imaging to biological tumor volumes (BTVs), defined by the uptake of ^18^F fluoroethyltyrosine (FET) for the radiotherapy planning of high-grade glioma, using a dedicated positron emission tomography (PET)-CT scanner equipped with three triangulation lasers for patient positioning.

**Methods:**

Nineteen patients with malignant glioma were included into a prospective protocol using FET PET-CT for radiotherapy planning. To be eligible, patients had to present with residual disease after surgery. Planning was performed using the clinical target volume (CTV = GTV ∪ BTV) and planning target volume (PTV = CTV + 20 mm). First, the interrater reliability for BTV delineation was assessed among three observers. Second, the BTV and GTV were quantified and compared. Finally, the geometrical relationships between GTV and BTV were assessed.

**Results:**

Interrater agreement for BTV delineation was excellent (intraclass correlation coefficient 0.9). Although, BTVs and GTVs were not significantly different (*p *= 0.9), CTVs (mean 57.8 ± 30.4 cm^3^) were significantly larger than BTVs (mean 42.1 ± 24.4 cm^3^; *p *< 0.01) or GTVs (mean 38.7 ± 25.7 cm^3^; *p *< 0.01). In 13 (68%) and 6 (32%) of 19 patients, FET uptake extended ≥ 10 and 20 mm from the margin of the gadolinium enhancement.

**Conclusion:**

Using FET, the interrater reliability had excellent agreement for BTV delineation. With FET PET-CT planning, the size and geometrical location of GTVs and BTVs differed in a majority of patients.

## Background

Positron emission tomotherapy (PET) is used in neuro-oncology practice essentially for diagnosis[1,2], prognosis evaluation[3], staging procedures[4] and monitoring the tumor response after treatment[5]. It can also be used for planning purposes, as to combine the biological and morphological information to guide radiation dose delivery. As such, biologically image-guided radiation therapy (RT), coupled to the current anatomical imaging technology, will deliver optimally radiation, with a high-degree of geometrical precision and biological conformity.

High-precision radiation therapy necessitates however precise anatomical and biological target delineation. [(18)F]fluoroethyltyrosine (FET) has been shown to have a high sensitivity (>90%) and specificity (>80%) for glioma[6]. In vitro and in vivo experiments have demonstrated that FET enters the cell by specific amino acids transports, but is not incorporated into proteins [7-9]. The delineation of the glial tumor extent is easier with radiolabelled amino acids than with 18F-fluorodeoxyglucose (FDG)[10], as a result of the high glucose metabolism in the cerebral cortex of the latter tracer and is thus the rational for the integration of FET in glioma volume delineation for RT planning. Although inter-observer variability has been assessed for tumor definition with other amino-acids, no such analysis has been taken in glioma delineation with FET. If the tumour delineation with FET proves to be unreliable, the consequential treatment plans may be inappropriate. As such, the inter-observer variability of FET during the planning process must be thoroughly evaluated.

Defining biological target volumes (BTVs) can result in substantial changes of target volumes for the planning of RT, as the size and location of FET is defined by metabolic activity rather than by the morphologic process of glioma growth, defined by magnetic resonance imaging (MRI)[11,12]. This may consequently lead to larger radiotherapy fields that will irradiate a larger volume of brain and possible increase of acute or late adverse events. It is therefore of paramount importance to determine whether FET can be used to delineate glioma for radiation therapy and how this method compares to more traditional methods, such as conventional gross tumour volumes (GTVs) delineation using MRI.

The purpose of this study was 1) to assess the interrater variability of high-grade glioma delineation using FET; 2) to quantify the BTVs and GTVs and to assess their volumetric and geometric relationships and 3) to assess the treatment characteristics after FET PET RT planning.

## Methods

### Patients

The study population comprised 19 patients (10 females, 9 males), referred to Geneva University Hospital, who were prospectively entered into a protocol assessing the value of postoperative FET-PET imaging for the RT planning of high-grade glioma. The inclusion criteria for the trial were: 1) the diagnosis of high-grade glioma; 2) residual tumor on MRI performed ≤ 24 hours postoperatively; 3) Karnofsky performance status ≥ 70; 4) age between 18 years and 70 years; and 5) written informed consent. The patient's and tumor's characteristics are detailed in Table 1. Patients undergoing stereotactic biopsy were eligible. Patients presenting *de novo *or recurrent high-grade glioma were eligible for this study. No previous RT to the brain or meninges interfering with the protocol treatment plan was however allowed for the latter patients. Postoperative treatment consisted of RT, using a linac with multileaf collimation (Varian 2100 CD, Palo Alto, CA), and concomitant temozolomide, followed by adjuvant temozolomide for all patients[13]. This study was approved by the institutional ethical committee. All subjects gave written informed consent for their participation in the study.

**Table 1:** Patient characteristics (*n *= 19)

Characteristics	*n *(%)
Gender	

Female	10(53)

Male	9(47)

	

Age (years)	

Median	53.5

Range	20.9 – 75.0

	

Karnofsky performance status	

70	5(26)

80	7(37)

90	3(16)

100	4(21)

	

Type of surgery	

Biopsy	10(52)

Subtotal removal	8(42)

Gross total removal	0(0)

None*	1(5.5)

	

Histology	

Glioma, grade WHO IV*	14(74)

Glioma, grade WHO III	5(26)

	

MiB1 (%) *n *= 16	

Median	15

Range	5 – 50

	

Glioma	

*De novo *presentation (primary)	17(89)

Secondary	2(11)

	

Localisation	

Frontal	10(52)

Temporal	4(21)

Parietal	3(16)

Thalamus	1(5.5)

Brainstem*	1(5.5)

*Heterogeneous brainstem mass in T1-weighted MRI images, with a subtle rim of peripheral enhancement after gadolinium enhancement, considered a grade IV glioma in one patient

### PET-CT scan

Patients underwent subtotal resection or stereotactic biopsy (Table 1) and brain FET PET/CT imaging postoperatively (mean, 8.3 days) (Biograph 16; Siemens Medical Solutions, Erlangen, Germany) using listmode PET data acquisition at the Department of Nuclear Medicine between July 2006 and December 2007. One accrued patient presented with a heterogeneous brainstem mass in T1-weighted MRI images, with a subtle rim of peripheral enhancement after gadolinium enhancement, which was considered a grade IV glioma (Table 1). FET was prepared at the cyclotron unit of the University Hospital of Zürich. Patients were placed in scanning position and CT imaging was performed (120 kVp, 90 mAs, 16 × 1.5 collimation, a pitch of 0.8 and a 0.5 second rotation) with an individualized immobilization plastic mask. Patients were injected intravenously with 200 MBq of FET after a 4–6-h fasting period. The PET data acquisition was started immediately after tracer injection[14] and was collected in list-mode format to allow flexible choice of frames. The dynamic studies (3 × 10 minutes) corresponding to 1 bed position, were covering the head up to the second cervical vertebral body. Following Fourier rebinning and model-based scatter correction, PET images were reconstructed using two-dimensional iterative normalized attenuation-weighted ordered subsets expectation maximization[15]. The CT-based attenuation correction map was used to reconstruct the emission data. The default parameters used were ordered subsets expectation maximization iterative reconstruction with four iterations and eight subsets followed by a post-processing Gaussian filter (kernel full-with half-maximal height, 5 mm).

A set of three triangulation lasers (central and laterals) identical to those used on the linear accelerators were used for patient accurate positioning. Two-mm thick CT images were acquired for planning purposes.

### Magnetic resonance imaging/CT fusion

Patient's diagnostic 1.5 Tesla MRI (Gyroscan Intera, Philips, Cleveland, OH) studies (axial T1-weighted with gadolinium enhancement) were transferred through the hospital picture archiving communication system (PACS) to the virtual simulation workstation (AcQSim^® ^System, Philips Medical System, Cleveland OH) and were fused with the CT performed during the metabolic imaging. The head was not immobilized during the preoperative MRI examination. Acquisition was done using a standard head coil from the second cervical vertebral body upwards. Patients' CT and MRI were automatically fused according to the bony and non-bony anatomy (orbital cavity, clivus, nasal cavity, mastoid air cells, and optic nerve). The data from the postoperative MRI, performed within 24 hours on the same MRI unit, was not fused with the planning CT but these data (T1- [with gadolinium] and T2- weighted sequence) were used mainly to assess the extend of resection and any residual disease was comprehensively included during the GTV delineation for any residual disease.

### Biological and morphological gross tumor volume delineation

First, BTVs, as conceptualized by Ling *et al *[16], were independently contoured by 3 experienced radiation oncologists (D.C.W, HV and T.Z), one with nuclear medicine training (H.V), using the Leonardo^® ^platform (Siemens Medical Solutions/CTI, Knoxville, TN). All brain CT images were interpreted by an experienced diagnostic radiologist. The PET, CT, and fused PET/CT images were displayed for review in axial, coronal, and sagital planes. All studies were interpreted and reviewed with knowledge of the patient's clinical history and results of previous imaging studies. The biopsied tumor, or residual tumor, defined by FET uptake was delineated manually. Maximum standardized uptake values (SUV_max_) were calculated for ROIs of focal hyperactivity by dividing the observed activity per gram in attenuation corrected PET with the injected activity per gram body weight[17]. A threshold value of 40% of SUV_max _was considered for the tumor margin in all patients, as FET tumor/brain uptake ratio may be inappropriate in high-grade glioma patients[14]. This value was determined previously in a set of high-grade glioma patients in a delineation comparative study, as the best thresholding value discriminating optimally the tumoral and background (grey matter in the opposite hemisphere) maximum SUV[18].

In the delineation process, the same windowing was used. Each physician (DCW, HV and TZ) manually delineated the BTVs. Within the Eclipse treatment planning station (TPS), composite, common and differential BTVs were generated using a Boolean algorithm. Common BTV (_COMMON_BTV) were defined as the intersection of all observers' BTVs (_COMMON_BTV = BTV_DCW _∩ BTV_HV _∩ BTV_TZ_). Finally, differential BTVs were defined as: _DIFF_BTV = [BTV_DCW _∪ BTV_HV _∪ BTV_TZ_] - [BTV_DCW _∩ BTV_HV _∩ BTV_TZ_]. Observer's BTVs, _COMMON_BTVs and _DIFF_BTVs were transferred to the AcQSim^® ^workstation using the PACS for planning purposes. A BTV-interrater agreement was assessed by intraclass correlation coefficient (ICC) computations[19].

Second, gross tumor volume (GTV) was defined as the residual macroscopic tumor after surgery or biopsy and the preoperative GTV (gadolinium ring contrast enhancement). GTVs were defined by one radiation oncologist (D.C.W) in the AcQSim^® ^virtual simulation workstation.

### Comparative assessment of the metabolic- and morphologic tumor volumes

The GTV data was also transferred from the AcQSim^® ^workstation to the Eclipse^® ^(Varian Medical Systems, Palo Alto, CA) TPS, for volume analysis and volumetric comparison. The selected BTV for comparison was defined by one radiation oncologist (D.C.W) for consistency. Clinical target volume (CTV) was defined as the union of the GTV and BTV (CTV = GTV ∪ BTV). Noteworthy, CTV defined the volume of microscopic spread but was not defined as per the ICRU formalism in this prospective protocol. The common volume between the GTVs and BTVs was also assessed (_COMMON_CTV = GTV ∩ BTV). Additionally the differential CTV (_DIFF_CTV = [GTV ∪ BTV] - [GTV ∩ BTV]) was computed. The Boolean operator in the Eclipse^® ^TPS was used for volume measurements and volume mismatch analysis. In case of BTV/GTV mismatch, the differential margins of these two volumes were measured on axial slices.

### RT planning

For treatment planning, the MD Anderson Cancer Center target policy was used[20]. Planning was performed on the CTVs. The planning target volume (PTV) included the CTV plus an anisotropic margin of 20 mm, not including however comprehensively the T2-weighted sequence hyperintense signal seen on the postoperative MRI.

### Treatment characteristics with FET PET planning

As to determine the impact of FET PET-guided RT planning, the treatment characteristics of the study patients were retrospectively assessed. The treatment characteristics of 19 other matched high-grade glioma patients (tumor location, GTV) were also analyzed. The difference of all study and matched patient's GTV were less than 10%, except for a patient with a brainstem glioblastoma. For this case and his matched counterpart, GTVs were 4.5 and 2.2 cm^3^, respectively. Excluding this latter patient, the median percentage-difference between the study and matched patients was 0.7% (range, -7.3 – 8.4).

### Statistical analysis

We performed all analyses using the Statistical Package for Social Sciences (SPSS, Ver. 15.1, SPSS Inc, Chicago, IL). For descriptive analyses of patients' characteristics and volumes sizes we used percents and mean score. The GTV, BTV and CTV delineation methods were compared using the Wilcoxon signed-rank test as numerical data were not normally distributed. The field size comparisons of the FET PET-guided- and non-FET PET-guided RT were performed using the Man Whitney U test. Statistical analyses used to test the interrater reliability of the biological tumor volume delineation by the three observers were the intraclass correlation coefficient and analysis of variance with the expectation to uphold the null hypothesis[19]. A two-sided random effect model was used. A *p *value of less than 0.05 was considered to indicate statistical significance.

## Results

**Figure 1:**
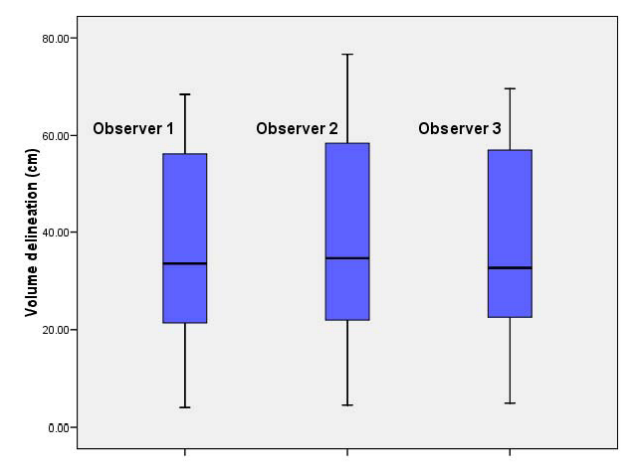
**Biological tumor volume measurements by three observers for each high-grade glioma case (1 through 19)**.

Abnormal FET uptake was observed in all patients. Median SUV_max _at 0 – 10, 10 – 20, 20 – 30 minutes were 3.05 (range, 0.51 – 4.52), 3.64 (range, 1.6 – 6.31) and 3.77 (range, 1.91 – 7.22), respectively. Fig. 1 details the BTV contoured by each observer. Mean BTVs for observer 1, 2 and 3 were 35.8 ± 21.7, 39.1 ± 23.6 and 36.3 ± 21.8 cm^3^, respectively. The interrater agreement was excellent (ICC = 0.9) and volumetric difference between observer's BTV delineation did not reach statistical significance (p = 0.99). The mean _COMMON_BTV was 32.0 ± 20.1 cm^3^. The _DIFF_BTV ranged from 0.3 to 19.0 cm^3 ^(mean, 8.0 ± 5.3).

The results of volumetric measurements of GTV, BTV and CTV are presented in Table 2. The BTVs were usually larger, but not significantly so (*p *= 0.9) than their morphologic counterpart: mean BTV and GTV were 35.8 ± 21.7 and 38.4 ± 25.7 cm^3^, respectively (Table 2).

**Table 2:** Measurements of tumor volumes in 19 patients with high-grade gliomas.

Pt. No.	BTV	GTV	CTV	_COMMON_CTV	_DIFF_CTV
	(cm^3^)	(cm^3^)	(cm^3^)	(cm^3^)	(cm^3^)

					

1	26.1	30.1	51.3	16.1	34.3

2	61.6	59.9	85.0	52.6	29.7

3	58.6	35.3	78.0	29.6	48.1

4	12.4	24.9	40.6	11.1	28.3

5	65.1	37.2	73.7	36.2	35.6

6	53.8	50.0	66.5	44.9	20.7

7	34.6	22.2	45.4	14.0	30.8

8	62.8	41.2	68.1	41.2	24.4

9	68.5	73.4	120.2	30.7	88.1

10	52.3	63.1	84.4	37.5	43.4

11	22.0	28.8	39.8	14.5	23.6

12	33.7	15.4	40.1	10.7	28.6

13	20.8	113.0	117.6	19.0	98.3

14	26.6	24.0	36.1	22.8	13.2

15	3.9	5.0	10.1	0.7	9.1

16	32.7	39.9	48.5	27.2	19.4

17	6.2	39.0	42.8	2.9	39.3

18	33.7	22.1	40.7	19.6	19.5

19	5.0	4.5	8.8	1.1	7.6

**Figure 2:**
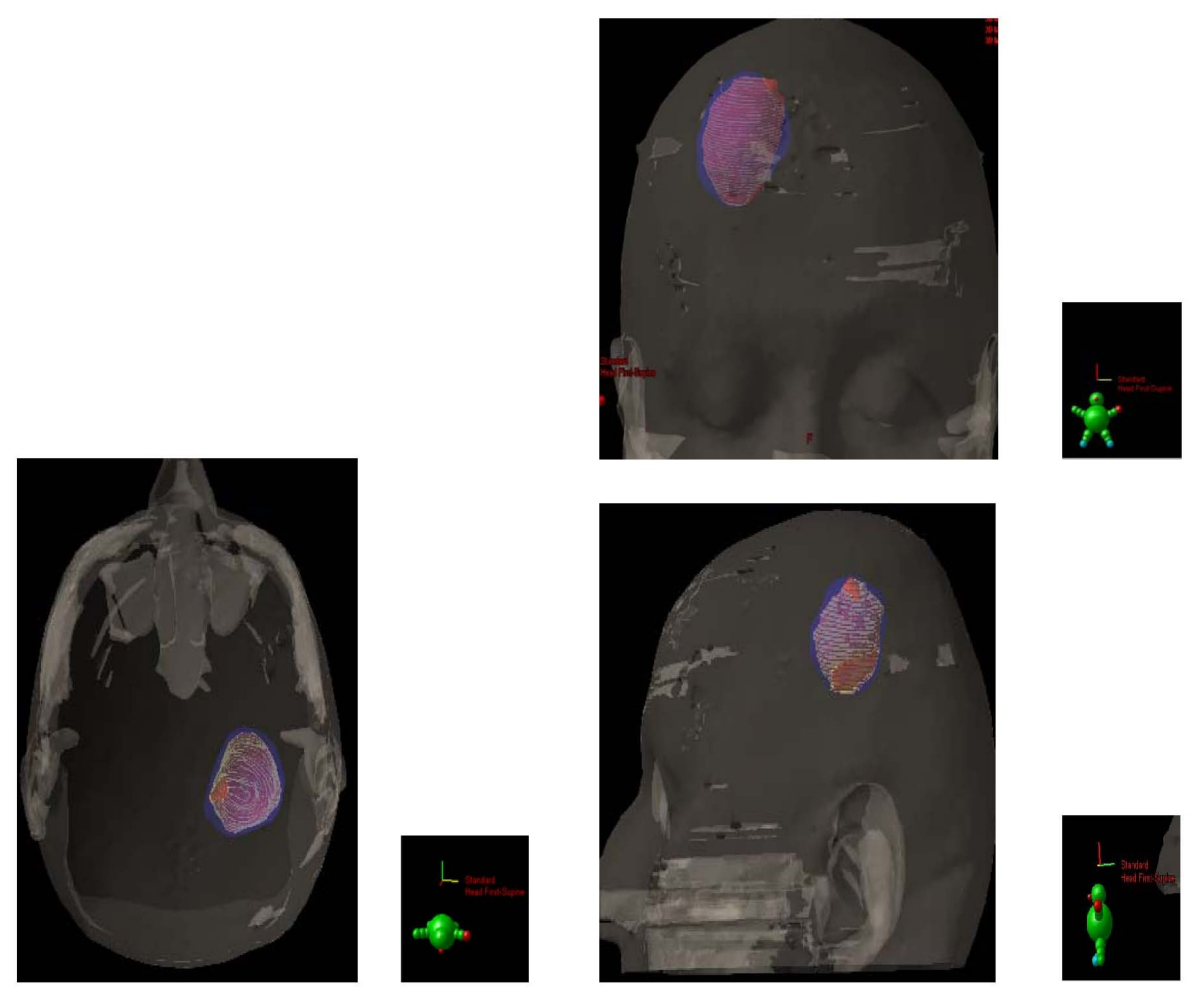
**Biological (BTV, blue) and morphological gross tumour (GTV, red) volume defining the clinical target volume in 19 patients with high-grade glioma**. Note the common volume between the tumour volumes (yellow chicken wire). Good BTV-GTV matching is shown in 1 patient.

**Figure 3:**
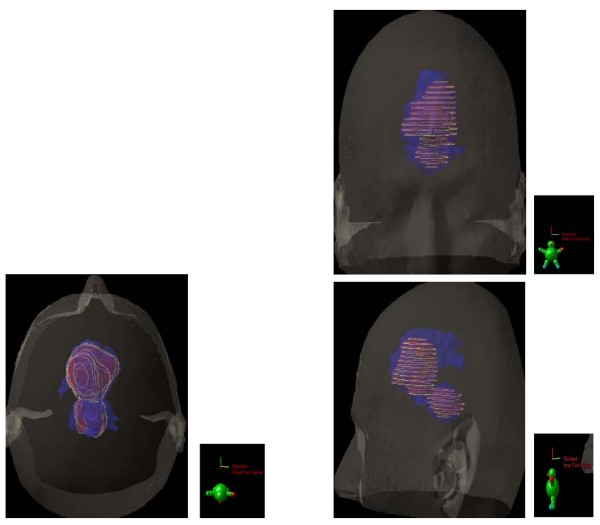
**substantial BTV-GTV mismatch is also detailed in 2 other patients**.

**Figure 4:**
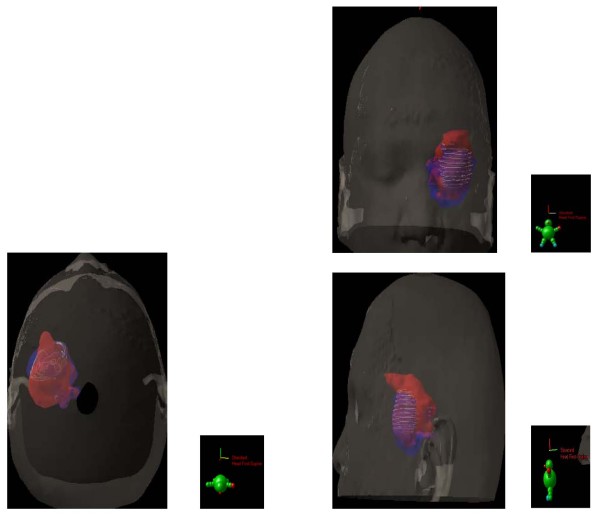
**substantial BTV-GTV mismatch is also detailed in 2 other patients**.

Unsurprisingly, the CTV, with which the patients were planned, were significantly larger than the GTV (*p *< 0.01) or the BTV (*p *< 0.01). For the whole group, the mean CTV was 57.8 ± 30.4 cm^3^and the mean _COMMON_CTV was 22.8 ± 15.1 cm^3 ^(Table 2). The _DIFF_CTV ranged from to 7.6 to 98.3 cm^3 ^(mean 33.8 ± 23.6; Table 2). The mean ratio (_COMMON_CTV)/(CTV) was 37.3% and ranged from 6.8% to 67.5%, indicating a mismatch in a substantial number of patients. FET uptake was detected up to 34.8 mm beyond gadolinium enhancement (mean, 15.1 ± 8.1 mm; range, 4.6 – 34.8) in 1 patient. The mean BTV located outside the GTV was 18.3 ± 12.4 cm^3 ^and ranged from 3.2 to 45.5. Thus, the percentage of BTV not included in the GTV ranged from 3.9% to 155.2% (mean, 62.6%), bearing in mind that occasionally the BTV was larger than the GTV. In 13 (68%) and 6 (32%) of 19 patients, FET uptake extended = 10 and 20 mm from the margin of the gadolinium enhancement. Likewise, gadolinium enhancement was detected up to 35.9 mm beyond FET uptake (mean, 13.4 ± 9.6 mm; range, 0.0 – 35.9) in 1 patient. The mean GTV located outside the BTV was 15.0 ± 22.3 cm^3^and ranged from 0.0 to 93.8. In 12 (63%) and 4 (21%) of 19 patients, gadolinium enhancement extended = 10 and 20 mm from the margin of the FET uptake. The target volumes are presented in Fig 2, with a relevant case presenting a good BTV-GTV matching (Patient # 14, Table 2; Fig. 2). Target volumes of 2 other patients are detailed, presenting with FET uptake located beyond the gadolinium enhancement (Patient # 8, Table 2; Fig. 3) and gadolinium enhancement located beyond the FET uptake (Patient # 1, Table 2; Fig. 4), respectively.

The mean number of treatment fields for the FET PET-guided- and non-FET PET-guided RT were 2.5 (range, 2 – 3) and 2.6 (range, 2 – 4), respectively. The size of the lateral (median, 9.6 *vs*. 9.1 cm; *p *= 0.83 and 9.5 *vs*. 8.2 cm; *p *= 0.37) and axial treatment fields (median, 9.6 *vs*. 8.8 cm; *p *= 0.33 and 9.5 *vs*. 8.9 cm; *p *= 0.37) of FET PET-guided and non-FET PET-guided RT were not significantly different.

## Discussion

For this prospective study, the choice of FET was dictated by its easy biosynthesis, *in vivo *stability and wide clinical distribution[9,21]. With an ^18^F-109 minutes half-life, FET-PET scanning is possible in centers without an in house cyclotron facility, and thus makes this tracer ideal for brain imaging in oncology. It is also hypothesized that FET may be superior to MET, as the former tracer in animal models exhibits no uptake in inflammatory cells, cerebral abscess and lymph nodes, showing potentially a higher specificity for the detection of cancer cells [22-24]. In a clinical setting, these two tracers can be however equally used. Weber *et al*. reporting on 16 brain tumor patients observed that the contrast between tumor and brain was not significantly different between MET and FET and that MET and FET uptake correlated well (r = 0.98), although the tracer's kinetics were indeed different[25]. Using FET to define the target volume for conformal RT necessitates however that the use of this radiolabeled amino acid for tumor delineation is reproducible and thus that the interobserver variability during this process is minimal. Van Laere *et al*. reported on 30 patients with suspected recurrent primary brain tumors[26]. A direct comparison of FDG and MET-PET was performed and the inter-observer agreement was assessed. It was 100% for MET and 73% for FDG. Our data are in keeping with these results, as the interrater correlation during target delineation using FET was excellent (ICC = 0.9; Fig. 1) and enabled the observer to define the BTV, using the selected SUV_max _threshold value, appropriately.

In their seminal paper, Hochberg *et al*. have described the propensity of malignant cell to invade the peritumoral edema or normal-appearing brain parenchyma. In 35 GBM untreated cases, 29 (> 80%) showed postmortem macro- and microscopic tumor invasion within a 2-cm margin of the tumor visualized by CT scan[27]. MRI has provided an incremental advance in high-grade glioma imaging. Several series have shown undisputedly that tumor infiltration, proven with stereotactic biopsies, was identified in areas congruent with abnormal signal on MRI images[28,29]. In a biopsy-controlled glioma study, MET and FET improved the tumor extension delineation by the combined use of FET-PET and MRI or CT, in comparison with conventional imaging alone[30,31]. We are presently left with the question of how to integrate optimally these various imaging modalities for tumor delineation. Grosu *et al*. reporting on 39 resected high-grade patients have shown that only a minority of patients (13%) had a good morphological and biological tumor volume concordance[11]. Moreover, a substantial mismatch between these two volumes was observed: one patient out of two had MET uptake extension beyond the hyperintensity signal on T2-weigted MRI. Likewise, gadolinium extension was observed outside the MET uptake in a majority (69%) of patients. This morphological and biological non-conformity has been observed in other series[12] and is in line with our results (Table 2; Fig. 3 and 4). Consequentially to these published results, the CTV was prospectively defined as the union of both BTV and GTV in this protocol. According to the ICRU definition, CTV should include all region of possible microscopic spread. Using a biologic paradigm, we believe that this region may be best defined by the summation of the morphological and biological data and not by a generically-defined 3D margin. In our series, the region of FET uptake beyond 20 mm of the Gadolinium enhancement in one third of patients is however remarkable. In short, this observation suggests that in a substantial number of patients the current RT margins may not be appropriate. This aforementioned consideration should be however validated in the follow-up of this study. Plan is to fuse the PD-volumes with the target volumes (i.e. BTV, GTV and CTV).

In our study, the BTVs were usually larger than their morphologic counterpart (Table 2). This observation is in line with the German data, which showed that the MET PET volumes were also larger than the ones defined by gadolinium enhancement (19.0 *vs*. 11.0 cm^3^). Unlike the aforementioned data, Mahasittiwat *et al*. reported smaller MET PET- (mean, 6.4 cm^3^), when compared to gadolinium-defined (mean, 92.1 cm^3^), tumor volumes[12]. The definition of the amino acid threshold SUV value may partially explain this discrepancy, although the extent of surgery could also be a factor. A delineation-threshold value of 1.7 for the tumor/normal tissue index was used in both studies. We used a defined percentage of the SUV_max_. Our group has investigated various strategies for FET-PET high-grade tumor delineation (Vees H, personal communication) using various functional image segmentation algorithms. This data will be published shortly. It remains to be determined which segmentation technique is the most appropriate for glioma delineation, further research using amino acid for tumor definition is justified in the framework of prospective protocols.

The use of the combined BTV and GTV for the FET-guided RT planning resulted in a non-significant increase of the fields' sizes. The FET-guided RT was however equitoxic to non-FET-guided RT, as none of the patients presented with CTCAE grade > 2 acute treatment morbidity (data not shown). It is somewhat paradoxical that the introduction of newer imagery modality, such as FET-PET, would translate into an enlargement of field size, as a result of the target volume increase. Planning techniques for RT in high-grade glioma patients, relying on CT or MRI for target delineation, usually result in a reduction in PTV[32,33]. As mentioned earlier, our group is currently following prospectively the patients from the current study, as to define precisely where the tumoral progression is located, relative to the BTV, GTV and CTVs. Plan is to import the diagnostic MRI performed for tumor progression into our TPS and to assess the BTV and recurrent tumor volumetric and geometric relationships. Ultimately, if the tumor progression is indeed documented in the BTV in a majority of cases, it may be advantageous to administer a simultaneous integrated boost (SIB) to the BTV, as dose escalation may have a possible effect on survival as shown in mathematical models using Monte Carlo simulations[34,35]. Several historical and contemporary series have shown however that dose escalation above 60 Gy, using non-metabolic target volumes, does not result in improved survival but causes, more often than not, more adverse events [36-39]. Moreover, the failure pattern analysis of high-grade glioma treated with high-dose (> 80 Gy) radiation indicates generally a predominant local pattern, suggesting that the morphological-defined tumor volumes are indeed inappropriate[40]. Other series have reported a significant increase in out-field failures after high dose RT[41,42]. It is however unclear if this differential failure pattern results from dissimilar failure definitions or parameters related to RT techniques or surgery. Boosting the radiation dose to a limited volume containing [34,35] tumor cells, not identified by non-metabolic imaging, may be highly desirable, using amino acids. The SIB paradigm has been successfully applied in a small Japanese series, delivering 68 Gy hypofractionated RT to the GTV, defined as the area of intensive MET uptake[5].

## Conclusion

Using a threshold value of 40% of FET SUV_max _for BTV delineation, the interrater delineation was excellent. FET PET- and MRI-defined tumor volumes differed substantially. In our series, only a minority (5%) of patients had good BTV and GTV concordance. The RT planning for high-grade glioma, based on a biologic paradigm, has shown a non significant treatment field increase, when compared to conventionally (i.e. GTV based on MRI enhancement) planned RT.

## Abbreviations

PET: Positron emission tomotherapy; RT: radiotherapy; FET: [(18)F]fluoroethyltyrosine; FDG: 18F-fluorodeoxyglucose; BTV: biological target volume; MRI: magnetic resonance imaging; GTV: gross tumour volume; PTV: planning target volume. CTV: Clinical tumor volume; _COMMON_CTV: Common clinical tumor volume; _DIFF_CTV: Differential clinical tumor volume; WHO: World Health Organisation.

## Competing interests

The authors declare that they have no competing interests.

## Authors' contributions

DCW was responsible for the primary concept and the design of the study; DCW, TZ, NC and HJV performed the data capture and analysis. DCW drafted the manuscript; GH performed the statistical analysis; DCW and TZ reviewed patient data; FB, HZ, RO and RM revised the manuscript.
